# Insanity in Chicago

**Published:** 1883-11

**Authors:** S. V. Clevenger

**Affiliations:** Special Pathologist Cook County Insane Asylum; Professor of Anatomy, Art Institute, Chicago, etc.


CHICAGO MEDICAL

JOURNAL & EXAMINER.

Vol. XLVII.—NOVEMBER, 1883.—No. 5.

### Original Communications.

Article I.

Insanity in Chicago. By S. V. Clevenger, m.d., Special Pathologist Cook County Insane Asylum; Professor of Anatomy, Art Institute, Chicago, etc. Read before the Chicago Medical Society, October 15, 1883.

While collecting the material for this article, it was my intention to present an exact history of the medical progress of this asylum from its inauguration, in addition to the statistical information given herein, but many things interposed to baffle my endeavors, prominent among them being the great Chicago fire, which destroyed all the county records, and the paucity of asylum papers dating earlier than 1878, in which year the present medical superintendent, Dr. J. C. Spray, assumed charge. He found the asylum books kept in a very careless manner; blotted, smeared, and full of errors. He transferred to new pages all the case histories obtainable from what little there was of previous record, and sought among the friends of such patients as were in the asylum for fuller details. These, as written down by him and his assistant physician, were turned over to me upon my appointment, and greatly pleased was I at the mass of information, in addition to the routine records, I was enabled to transcribe into large new case books, the memoranda of which had been laboriously and systematically made by these gentlemen. But this information mainly concerns those patients who were admitted during Dr. Spray's accession to the superintendency, and thus, in analyzing cases earlier than 1878, many sources of error must be eliminated before they can be made available statistically. There is one patient in the asylum who is said to have entered in the year 1857 (she enjoys the pseudonym of Katy Darling). The records assert her to have been admitted in 1866, but as there was no asylum here in either year, and as no attempt was made to keep even the semblance of a record of patients until Dr. Tope's appointment in 1870, it isn't worth while fussing about a little matter of nine or ten years in a patient's career under such circumstances. In *ante bellum* days there was no county asylum. It seems that, by some means or other, several insane patients began to accumulate in the county poor-house, near Jefferson, Illinois, and I am informed by Dr. Theo. H. Patterson, who treated the county patients in 1866, that in that year there were between 20 and 30 insane persons in a single ward of the poor-house. As will be seen in the tabular part of this paper, Dr. Patterson's statement is quite reasonable. Drs. Webber and Kielher succeeded Dr. Patterson, and from October 1, 1867, to January 10, 1871, Dr. D. B. Fonda had charge of the poor-house as medical visitor. Some very comical stories are told of this *regime.* It seems that a warden was the great man over paupers, sane and insane, in those days, and upon one occasion a grand jury insinuated its obnoxious presence into the poor-house. A certain member of the jury ventured to ask the ruling potentate for his accounts. "Accounts," vociferated the warden, "Of course I keeps 'em. He pulled a child's copy-book from his pocket ; another story has it he found it after much ransacking in the buildings), and held it up indignantly for inspection. "Here they comes in and there they goes out!" he shrieked. Whether he alluded to money or patients, or both, I have never been able to ascertain, but as at this date the warden, and not the doctor, was the much more important personage, it is presumable his showing was financial, and not medical. The welfare of a patient was insignificant alongside the chances of stealing a dollar or so in those times. Dr. John W. Tope was appointed medical superintendent January 1, 1871. By this time, the poor-house had outgrown itself, and the necessity for differentiating insanity from pauperism had become apparent enough to justify the erection of a large brick building capable of accommodating 300 patients (at this writing it has 519 within its walls, and more arriving weekly). Be it said to Dr. Tope's credit, that he started out with true medical *animus*, to better the condition of the insane, but, judging from the growing carelessness of the records, he or his successor, I am unable to ascertain which, became discouraged under the incessant fire of political bummers, and turned the bookkeeping over to the cranks.

Dr. Cunningham was appointed January 1, 1875, and served until Dr. Spray's term began, January 1, 1878. The year following I found this gentleman struggling against great difficulties while seeking the elevation of the medical management to a respectable status. He afforded me every facility possible for investigation. In seeking to benefit the living we have held autopsies at a time when the bare fact of his purchase of a post mortem case of instruments was used against him by such enemies as alway beset the path of those who have done good work in the world.

Medical men in European asylums have advanced our knowledge and treatment of insanity, not because they are intellectually superior to American physicians, but because in Europe medicine is afforded all the assistance necessary for its advancement, while on this side the Atlantic the medical superintendent is harrassed and embarrassed in innumerable ways, by all sorts of people, about all sorts of matters absolutely foreign to what should be his legitimate duties. There seems to be no relief ahead until the general public shall take a more enlightened interest in the insane, and strengthen the hands of the honest superintendents of asylums, instead of contenting themselves with the vulgar belief that every institution of the kind is a hell under any circumstances. The people listen to the idle or malicious stories set afloat by designing place-hunters; but while ever ready to condemn, will not sustain, by a simple investigation and endorsement, the management which has been unjustly assailed.

The earliest authentic record in my care relating to the inmates, is a verdict signed and sealed by the County court clerk, dated April 30, 1867, which sets forth the fact that on that day the County court convened at the Cook county Hospital for the Insane, and found twenty-seven males and forty-two females named in the verdict insane. The erroneous condition of the records is at once apparent upon comparing the number given by the verdict as present in 1867 with the list of admissions entered on the books previous to and including 1867 in this table.

### TABLE I.

ADMISSIONS.

| Year. | Males. | Females. | Total. |
|---|---|---|---|
| 1860 | 1 | ... | 1 |
| 1861 | ... | ... | ... |
| 1862 | ... | ... | ... |
| 1863 | 1 | 1 | 2 |
| 1864 | 1 | 1 | 2 |
| 1865 | ... | ... | ... |
| 1866 | 6 | 12 | 18 |
| 1867 | 7 | 13 | 20 |
| 1868 | 7 | 6 | 13 |
| 1869 | 9 | 17 | 26 |
| 1870 | 28 | 24 | 52 |
| 1871 | 75 | 91 | 166 |
| 1872 | 71 | 43 | 114 |
| 1873 | 84 | 57 | 141 |
| 1874 | 83 | 73 | 156 |
| 1875 | 86 | 77 | 163 |
| 1876 | 126 | 110 | 236 |
| 1877 | 108 | 101 | 209 |
| 1878 | 104 | 105 | 209 |
| 1879 | 151 | 146 | 297 |
| 1830 | 134 | 130 | 264 |
| 1881 | 136 | 118 | 254 |
| 1882 | 199 | 153 | 352 |

It is evident from this discrepancy that there must have been a population of total admissions nearer 3,500 than the figures given.

### TABLE II.

DURATION OF RESIDENCE, IN ASYLUM, OF MALE PATIENTS.

| UNDER | 1 week. | 2 weeks. | 1 month. | 3 months. | 6 months. | 9 months. | 1 year. | 1½ years. | 2 years. | 3 years. | 4 years. | 5 years. | 6 years. | 7 years. | 8 years. | 9 years. | 10 years. | 11 years. | 16½ years. | Irregular. | Total. |
|---|---|---|---|---|---|---|---|---|---|---|---|---|---|---|---|---|---|---|---|---|---|
| Recovered | 2 | 9 | 21 | 56 | 34 | 13 | 8 | 6 | 6 | .. | 1 | .. | .. | .. | .. | 1 | .. | .. | .. | ... | 157 |
| Improved | 3 | 4 | 12 | 35 | 45 | 16 | .. | 12 | 7 | 5 | .. | 3 | 1 | 1 | 1 | 1 | 1 | .. | .. | 77 | 224 |
| Unimproved | 8 | 1 | 5 | 10 | 13 | 7 | 2 | 5 | 1 | 4 | 3 | 1 | 3 | 2 | 1 | 1 | .. | .. | .. | ... | 67 |
| Sent to State Hospital | 12 | 27 | 26 | 35 | 26 | 20 | 13 | 14 | 8 | 10 | 8 | 3 | 4 | 4 | 3 | 1 | .. | 1 | .. | 33 | 248 |
| Died | 11 | 13 | 19 | 51 | 35 | 20 | 12 | 18 | 16 | 24 | 11 | 16 | 6 | 6 | 4 | 3 | 2 | 4 | 1 | 33 | 305 |
| Unknown | .. | .. | .. | .. | ... | .. | .. | .. | .. | .. | .. | .. | .. | .. | .. | .. | .. | .. | .. | ... | 26 |
| Total | 36 | 54 | 83 | 187 | 153 | 76 | 35 | 55 | 38 | 43 | 23 | 23 | 14 | 13 | 9 | 7 | 3 | 5 | 1 | 143 | 1027 |

### TABLE III.

DURATION OF RESIDENCE IN ASYLUM OF FEMALE PATIENTS.

| | 1 week. | 2 weeks. | 1 month. | 3 months. | 6 months. | 9 months. | 1 year. | 1½ years. | 2 years. | 3 years. | 4 years. | 5 years. | 6 years. | 7 years. | 8 years. | 9 years. | 10 years. | 11 years. | 12 years. | 13 years. | 14 years. | 15 years. | 16 years. | 16½ y'rs. | Irregular. | Total. |
|---|---|---|---|---|---|---|---|---|---|---|---|---|---|---|---|---|---|---|---|---|---|---|---|---|---|---|
| Recovered | ... | 5 | 24 | 54 | 44 | 17 | 6 | 11 | 5 | 3 | 1 | 2 | ... | 1 | ... | ... | ... | ... | ... | ... | ... | ... | ... | ... | ..... | 173 |
| Improved | 4 | 5 | 12 | 24 | 26 | 13 | 8 | 5 | 7 | 7 | 1 | ... | 1 | 1 | 2 | ... | ... | ... | 1 | ... | ... | ... | ... | ... | 60 | 177 |
| Unimproved | 7 | 2 | 2 | 5 | 5 | ... | 4 | 2 | ... | 1 | 4 | 2 | ... | ... | ... | ... | ... | ... | ... | ... | ... | ... | ... | ... | ..... | 34 |
| Sent to State Hospital | 9 | 20 | 26 | 29 | 17 | 5 | 10 | 20 | 14 | 4 | 7 | 2 | 4 | 3 | 1 | ... | ... | 1 | 1 | 1 | 1 | ... | ... | ... | 6 | 181 |
| Died | 10 | 11 | 13 | 35 | 27 | 17 | 14 | 18 | 22 | 27 | 23 | 16 | 9 | 8 | 8 | 7 | 1 | 5 | 3 | 3 | ... | 1 | 1 | 1 | 35 | 315 |
| Unknown | ... | ... | ... | ... | ... | ... | ... | ... | ... | ... | ... | ... | ... | ... | ... | ... | ... | ... | ... | ... | ... | ... | ... | ... | ..... | 44 |
| | 30 | 43 | 77 | 147 | 119 | 52 | 32 | 56 | 48 | 42 | 36 | 22 | 14 | 13 | 11 | 7 | 1 | 6 | 5 | 4 | 1 | 1 | 1 | ... | 101 | 924 |

### TABLE IV.

| | MALE. | FEMALE. | TOTAL. |
|---|---|---|---|
| Recovered | 157 | 173 | 330 |
| Improved | 224 | 177 | 401 |
| Unimproved | 67 | 34 | 101 |
| Sent to State Hospital | 248 | 181 | 429 |
| Died | 305 | 315 | 620 |
| Unknown | 26 | 44 | 70 |
| Total | 1027 | 924 | 1951 |
| In Hospital | 237 | 282 | 519 |
| Total Individuals | 1264 | 1206 | 2470 |

For percentage, estimates, Table IV must be reconstructed by omitting all cases sent to State Hospitals for the insane and the unknown. By this procedure the percentage of deaths would be larger than it should be, for it is safe to presume that of the 70 unknown cases a minority died. We thus have an admission number available for calculation of 753 males and 699 females, resulting in the following showing per thousand :

TABLE V.

| | MALES. | FEMALES. | TOTAL. |
|---|---|---|---|
| Recovered | 208 | 247 | 227 |
| Improved | 297 | 253 | 276 |
| Unimproved | 89 | 48 | 69 |
| Died | 405 | 450 | 427 |

Which is putting the very worst phase possible forward. It is obviously unjust, for it is to be recollected that in Tables II and III 429 patients were sent to State Hospitals after a residence here of from one week to eleven years, and these asylums have the right to take from us selected patients, and *our best or most curable cases were taken from us by the State.*

On this ground I feel justified in including the State cases among the discharged improved in the next table. This, of course, lowers the death rate and raises the betterment list unduly, but, as in Table V, none of the insane present in the hospital were included by including them in the next calculation with the 70 unknown we have a basis of 2,470 individuals—1,264 males and 1,206 females which affords per 1,000.

TABLE VI.

| | MALES. | FEMALES. | TOTAL. |
|---|---|---|---|
| Recovered | 124 | 143 | 134 |
| Improved | 177 | 146 | 162 |
| Unimproved | 60 | 27 | 41 |
| To State Hospitals | 196 | 141 | 174 |
| Died | 241 | 262 | 251 |
| Unknown | 20 | 36 | 28 |
| In Hospital | 187 | 233 | 210 |

Should this appear to run to the other extreme owing to the impossibility of eliminating every discordant element, let us tabulate a mean between the two preceding estimates.

TABLE VII.

| | Males. | Females. | Total. |
|---|---|---|---|
| Recovered | 166 | 195 | 180 |
| Improved | 237 | 199 | 219 |
| Unimproved | 75 | 37 | 55 |
| Died | 323 | 356 | 339 |

There is much unnecessary cavil in the medical journals at the term "recovered" as used by asylum statisticians. If it honestly express the supposed condition at the time of the patient's discharge, no other word could be used instead. If a person actually recover from a recurring disorder of any kind whatsoever, the possibility of recurrence should not weigh against the present fact of actual, even though temporary, recovery. It is impossible for the asylum physician to follow even a small percentage of discharged patients to their death. Counting together the recovered, improved, and State Hospital cases as having at least undergone betterment by their stay in the asylum, the lowest, highest and mean estimates stand, then, as follows:

TABLE VIII.

MAXIMUM PER CENT.

| | Males. | Females. | Total. |
|---|---|---|---|
| Improved | 50.5 | 50.0 | 50.3 |
| Unimproved | 8.9 | 4.8 | 6.9 |
| Died | 40.5 | 45.0 | 45.0 |

TABLE IX.

MINIMUM PER CENT.

| | Males. | Females. | Total. |
|---|---|---|---|
| Improved | 49.7 | 43.0 | 47.0 |
| Unimproved | 6.0 | 2.7 | 4.3 |
| Died | 24.0 | 26.2 | 25.0 |

Let me parenthetically remark that where 100 cannot be obtained in footing up any table requiring it, a little study will reveal the reason therefor. Differently based calculations without all the factors.

TABLE X.

MEAN PER CENT.

| | Males. | Females. | Total. |
|---|---|---|---|
| Improved | 50.1 | 46.5 | 48.6 |
| Unimproved | 7.4 | 3.7 | 5.6 |
| Died | 32.2 | 35.6 | 33.8 |

Wm. Farr, M.D., C.B., D.C.L., Registrar General Statistician (whose researches were accredited to the doomed to be unknown head of that office), has recently published his English life tables, with the contained results of research from the year 1838 until just before his death in 1883. Forty five years experience as a patient monomaniac in statistics he indulged in, swallowing snubs of "superiors," devoting his entire life to the elucidation of problems of paramount interest to the real workers in the world, who wish to help the unthinking, reckless herd from the consequences of their own blunders. From his invaluable tables I learn that the mean duration of life, or mean after lifetime of males at birth, was 39.9 years, whereas by the new table it becomes 41.9, representing an increase of two years, or an addition of five per cent. to the mean duration of the lifetime of males. The recent addition to the lifetime of females appears to be still more marked. According to Dr. Farr's life table (see *British Medical Journal*, June, 1883), the mean duration of life of females was 41.9, whereas the new table makes it 45.3 years, representing the addition of nearly three years and a half, or more than eight per cent., to the average life-time of all females born. So far the paper proves that the reduced death rates signify an important addition to the lifetime both of males and females. Twenty to sixty he classes as the useful period of human life; other years are classed as dependent years. Thus, sixty-six per cent. of the increased duration of human life in England is lived at the useful and productive period, and not more than thirty-four per cent. at the dependent ages of childhood or old age. The British editor deduces that the increase of knowledge, with its opportunities for good work and intellectual enjoyment, are largely creditable with the increased duration of human life, and what can be more plausible than the supposition that when people grow more honest, less animal, and better acquainted with the conditions under which they live, they will survive as the fitter to live, by reason of their acquirements and living consistently with them.

The bearing of Farr's life table, placing the male climax at 41.9, and the female at 45.3, upon our present subject is this: Our last table shows the rapid increase of insanity after the thirtieth year, the greatest number of females being insane at thirty-five and males at forty, the mortality statement of Chicago city shows the maximum death rate there to be between the ages of twenty and forty; tallying with our showing of moribund mentality in the regions of those years. Just as it is not safe to draw general conclusions from the statistics of a single asylum, or even those of several asylums, so the ages at which somatic and mental death seems most likely to occur in Chicago, with its population of two-thirds of a million persons, for the most part mentally and physically overstrained, compared with Farr's climacteric ages, drawn from the vast population of Great Britain, largely composed of settled, steady routine workers, and rural drones, I look upon as indicating the driving, scrambling character of our environment with its necessary accompaniment of a shortened mental and bodily life. The *American Journal of Neurology and Psychiatry* for May, 1883, notes that Dr. Wilkins, of the California Asylum at Napa, prophesies from the rapid increase of insanity in California (1 to 833 in 1860, and 1 to 383 in 1880) that there will be one insane person to two sane in the year 2000 in that State. The *Journal* very properly remarks: It is obvious that Dr. Wilkins ignores the fact that California allures a large number of wandering lunatics and these, as the State increases in population, and the restraining influences of civilization assert itself, find themselves out of accord with their surroundings, are then recognized to be insane and committed to asylums.

This apparent resulting increase occurs in all new countries. Just so it is with the Chicago insane, the proportion of whom to the population is somewhere in the neighborhood of the California figures of 1880 (1 to 383). There is a great influx of paupers from Europe into Chicago. Indeed, whole parishes of paupers have been "assisted" by the English government hitherward and Chicago receives a good share of such assisted emigrants. There is evidence on the books to show that one case at least left the Christiania Asylum and sailed straightway across the sea to this institution. Basing proportions of insane to surrounding population requires more than the mere numerals.

Evelyn, in 1620, wrote of the grand and great climateric, the sixty-third year, and a popular notion has been that by muliplying seven into the odd numbers, three, five, seven, nine, critical ages are obtained. I do not find this supported by mortality statistics. Mr. John Mattocks, a prominent lawyer of Chicago, called my attention to the remarkable frequency of deaths at the age of sixty-three in proportion to the number of persons living at that age. I regret that my tables do not show the ages at which the insane have died in this institution. The time allotted me in which to finish this article will not enable me to make the necessary calculations.

Dr. Raynor, in the London *Lancet*, December 30, 1882, began an investigation, which led Dr. T. A. Chapman, the Medical Superintendent of the City and County Asylum, Hereford, England, to publish his statistical deductions on the "Recovery and Death Rates in Asylums," in the London *Journal of Mental Science*, April, 1883. Dr. Chapman bases his calculations upon the statistics of 701 English asylum years, dealing with 112,972 admissions, and 388,661 as an average number resident. His conclusions may be summarized thus:

1. English statistics show most favorable results with asylums of moderate size, holding 300 to 700 patients.

2. No two asylums in the world are capable of comparison together as to recoveries and death rates, for the circumstances governing each asylum make it *sui generis*. For instance, one asylum sends all terminal dements and hopeless cases to special institutions for them, and its death rate must necessarily be very small; another asylum takes only curable cases, etc.

3. An actively moving population affords the largest proportion of active disease, whether curable or fatal, and that the cost of such asylums is greatest.

4. Low death rates go with high recovery rates, and *vice versa*, notwithstanding that many individual asylums present statistics very much the other way.

Looking over the voluminous tables of Dr. Chapman, I can find nothing in the rates of recovery and deaths to enable me to make a comparison with our own figures.

The recovery rate is calculated on the total admissions, and covers a wide range, the average being 33.7 per cent.; but the death rate is based upon the average number resident, which I have been unable to imitate, owing to absence of records older than 1878. Both our recovery rate and death rate are figured from the total admissions, as far as attainable, and in the belief that omissions occurring in the old records balance themselves, I think our present estimate is close upon the truth.

Chicago is a rapidly-growing, progressive city, with a population of 600,000 or more, and invariably this asylum comes in for the very worst possible cases. The condition in which patients are often brought to this asylum is terrible, disgusting, and distressing. Our present State laws are largely to blame for this state of things. Patients are arrested, and pending trial are jailed in the old criminal building (the old Court House), often waiting there a week or more before trial. Dr. Bluthardt, the County Physician, is loaded down with business, and has insufficient means at his disposal to care for these wretches. The time will probably come when a place of detention will be given them, instead of incarcerating them in a common jail, but the entire insane law of Illinois needs vigorous overhauling. Opportunities are lost for improving the condition of patients in the beginning of the mental disorder, and every alienist knows that prompt treatment in the outset often averts chronicity.

An element well worth considering is that many deaths occur from just such intercurrent diseases as would have befallen these same patients had they been sane, such as Bright's disease and phthisis; also, that many figure in our death list who have, by reason of natural decline, as of old age, or the issue of a complaint existing long before admission. Glance along the line of deaths in the duration of residence table, and it will be seen that when a person lives here sixteen years or less and then dies, it is questionable if it be proper to place every such case to the charge of death from insanity, merely because the patient died while insane. The fact that the greatest percentage of death occurs in the first three months after admission, bears upon the acute condition of the patient's insanity or accompanying disease when brought here.

Dr. Spray and I have carefully eliminated from the statistics the irregular or readmitted cases which go to swell the rate of recoveries in many Eastern asylums, and lower the death rates.

For example, in the reports of a certain New York asylum, the death rate was amazingly low, and the recovery rate high. It was ascertained that the figuring had been done upon a fraudulent admisson number, by counting among the recoveries one man, for example, who had been repeatedly discharged recovered, and his 20 or more "cures" had been placed to the credit of the total cases recovered.

Dr. Alex. Thuemmler, who has been many years the efficient Assistant Physician at this asylum, tells me that to his personal knowledge, the St. Louis Asylum invariably turned over their dements to the poor-house, and in the year 1880, in consequence, reported only 15 deaths.

These political dodges are what causes so-called American Alienism to stink in the nostrils of European physicians interested in insanity statistics and treatment. The fact that it is probably the minority of American superintendents who resort to this sort of thing, does not help us at all. "*Mitgefangen, mitgehangen.*"

As far as ascertainable, the ages of patients upon their admission are given in the next table.

TABLE XI.

| AGES. | NO. OF MALES. | NO. OF FEMALES. | TOTAL. |
|---|---|---|---|
| 6 | 1 | ... | 1 |
| 13 | 2 | 2 | 4 |
| 14 | ... | 4 | 4 |
| 15 | 5 | 2 | 7 |
| 16 | 3 | 2 | 5 |
| 17 | 14 | 9 | 23 |
| 18 | 15 | 11 | 26 |
| 19 | 15 | 19 | 34 |
| 20 | 26 | 27 | 53 |
| To 25 | 203 | 194 | 397 |
| " 30 | 237 | 244 | 481 |
| " 35 | 238 | 241 | 479 |
| " 40 | 251 | 195 | 446 |
| " 45 | 167 | 119 | 286 |
| " 50 | 103 | 104 | 207 |
| " 55 | 69 | 86 | 155 |
| " 60 | 35 | 45 | 80 |
| " 65 | 24 | 22 | 46 |
| " 70 | 9 | 15 | 24 |
| " 75 | 5 | 11 | 16 |
| " 80 | 1 | 7 | 8 |
| " 85 | 1 | 2 | 3 |
| " 90 | ... | 2 | 2 |
| " 94 | ... | 1 | 1 |

The mistake must not be made here that this table gives the ages at which the patient became insane, because a large proportion was insane before arrival. It is the best showing we can make under the cirumstances, and is valuable as an index, at least, to the age at which the patient became troublesome to relations, or neighbors, or otherwise became "legally insane" which is far from meaning when he or she began to be medically insane.

Passing to a consideration of nativities, I wish I could afford the medical public as elaborate a disquisition as that of Dr. E. C. Spitzka's, published in the *American Journal of Nervous and Mental Disease*, October, 1880, constructed from the report of Dr. J. G. Kiernan, who was many years assistant physician at Ward's Island Asylum, New York. Dr. Spitzka's racial statistics run thus: Anglo-American, 438; English, 85; Scotch, 29—Anglo-Saxon Race, total, 552. German-Americans, 213; German, 268; Dutch, 11—Germanic Race, total, 492. Norse, 14; Swedes, 20; Danes, 11—Scandinavian Race, total, 45. Teutonic Family, total, 1089. Irish-American, 351; Irish, 332; Welsh, 16—Celtic Family, total, 699. Portuguese, 1; Spanish, 13; Italian, 20; French, 35—Latin Family, total, 69. Bohemian, 22; Polish, 2; Russian, 3—Sclavonic Family, total, 27. Hebrew, 204; Kurd, 1—Shemitic Family, total, 205. Chinese, 2; Hungarian, 2—Mongolian Family, total, 4. Negro Family, total, 204. Grand total 2,297. The records of that asylum also show the form of insanity, but careful diagnoses were not in order here in the days when "here they comes in and there they goes out," hence, I am debarred from sifting my records in this respect. When tables give unbalanced numbers it is because information is lacking in the old books, as for example in this instance, the nativities had not been ascertained in cases which I have been obliged to omit.

It is evident by the excess of these numbers over those in the total individual table, that the list is vitiated by about thirty re-admissions, but this does not affect the general result.

I would like to make a comparison of these figures with the census of Chicago, but I have too vivid a recollection of the worthlessness of census returns. I have seen the most abject stupids appointed to collect such data, and in one instance in the "Far West" I knew of one scrapegrace paid per capita who

NATIVITIES—TABLE XII.

| | Males. | Females. | Total. |
|---|---|---|---|
| America | 361 | 200 | 561 |
| Germany | 359 | 282 | 641 |
| Ireland | 327 | 362 | 689 |
| Norway | 62 | 29 | 91 |
| Sweden | 87 | 107 | 194 |
| Switzerland | 8 | 1 | 9 |
| France | 16 | 7 | 23 |
| Italy | 9 | 3 | 12 |
| Bohemia | 34 | 28 | 62 |
| Canada | 13 | 12 | 25 |
| Wales | 3 | 1 | 4 |
| Russia | 5 | ...... | 5 |
| Poland | 6 | 6 | 12 |
| " Colored " | 18 | 5 | 23 |
| England | 53 | 26 | 79 |
| Denmark | 11 | 1 | 12 |
| Holland | 11 | 2 | 13 |
| Scotland | 20 | 13 | 33 |
| Austria | 6 | 4 | 10 |
| Finland | 1 | ...... | 1 |
| Saxony | I | ...... | 1 |
| Hungary | 1 | ...... | 1 |
| Total | 1412 | 1089 | 2501 |

copied one thousand names from the Chicago Directory, accrediting them to his town for the sake of the pelf. Spitzka also calls attention to a similar difficulty in the census of New York, where many of the "takers" put down every one whose language they could not understand as a German.

As far as ascertained, the occupation of the insane males were: Laborers, 468; carpenters, 52; farmers, 52; clerks, 37; merchants, 33; tailors, 25; machinists, 23; painters, 20; printers and shoemakers, 17 each; blacksmiths and sailors, 16 each; bookkeepers, 12; cabinet-makers and peddlers, 13 each; watchmakers, 12; tinsmiths, bakers, liverymen, cigarmakers, 11 each; saloon-keepers, butchers, 10 each; plumbers, stone-cutters, 9 each; engineers, 8; clergymen, physicians, firemen, upholsterers, 7 each; salesmen, 6; showmen, vagrants, policemen, teamsters, telegraphers, waiters, bricklayers, apothecaries, 5 each; schoolteachers, porters, confectioners, barbers, musicians, street car conductors, engravers, expressmen, weavers, coopers, 4 each; gardeners, watchmen, plasterers, bookbinders, locksmiths, coachmen, soap-makers, millers, 3 each; artists, silver-platers, rag-pickers, moulders, lake captains, shorthand reporters, harness-makers, railroad cashiers, distillers, collectors, editors, showcase manufacturers, reporters, mirror-makers, schoolboys, errand boys, saddlers, hatters, messenger boys, stewards, boat-builders, stair-builders, tanners, cooks, house-movers, brakesmen, students, janitors, plow manufacturers, bill-posters, grocers, gas fitters, tobacconists, store-keepers, steam-fitters, 2 each; stamp manufacturer, wagon-maker, rope-maker, roofer, sewer builder, decoy duck maker, brewer, lithographer, carriage painter, "clairvoyant," instrument makers, knife-grinder, coppersmith, brass-finisher, railroad fireman, cattle dealer, civil engineer, inventor, basket-maker, lather, whitewasher, actor, glove-maker, pattern-maker, iron-worker, boiler-maker, auctioneer, attendant in insane asylum, razor manufacturer, cutler, caulker, drover, gold leaf manufacturer, polisher, detective, dyer, hotel runner, chiropodist, well-digger, "homœpath," switch-tender, glazier, newsboy, lawyer, locomotive engineer, locomotive fireman, contractor, nurseryman, boss of iron mill, child, 1 each.

During the preparation of the foregoing table many comical ideas arose to my mind suggested by the juxtaposition of incongruities, but as insanity is no joking matter, I shall leave my readers to their fun if disposed to see anything funny in generalizations such as the preceeding, while the physicians feel heart-sick over the scenes and particulars daily witnessed by them.

The vulgar idea that any especial trade or profession inures particularly to insanity should find dissipation after looking over the table preceeding. Avocation as a predisposing or exciting cause of insanity, finds no support from our statistics.

Table XIII.

OCCUPATIONS.

| | Males | Females | Total |
|---|---|---|---|
| *Unskilled:* | | | |
| Laborers | 469 | | |
| Housewives | | 770 | |
| Domestics | | 326 | |
| Peddlers | 115 | 5 | 1685 |
| *Skilled:* | | | |
| Mechanical | 302 | 60 | |
| Professional | 31 | 10 | |
| Mercantile | 65 | 4 | 472 |
| *None:* | | | |
| Children | 3 | 2 | |
| Paupers | | 13 | 18 |
| | 1190 | 985 | 2175 |

Female pursuits were as follows: Housewives, 762; servants, 319; seamstresses, 39; paupers, 12; dressmakers, housekeepers, tailoresses, each 8; salesmen, 4; cooks, fortune-tellers, school-teachers, washwomen, music teachers, 3 each; school-girls, milliners, governesses, prostitutes, 2 each; actress, nurse girl, vagrant, editress, farmer's daughter, cantatrice, 1 each.

The civil condition, as far as sought out, was: Males—single, 314; married, 366; widowed, 26. Females—single, 355; married, 579; widowed, 108.

Though the records are imperfect, the above named tend to militate against the idea of benedict immunity from craziness.

Since the foregoing was written Dr. Bluthardt informed me that during his service as one of the Board of supervisors, from 1869 to 1871, the intention was to have an asylum here for the incurable insane and a retention hospital.

The statistics of the Kankakee and Elgin Asylums, so far as they relate to Chicago insane, should form an integral part of future estimates in connection with our own, in arriving at an idea of the proportion of insane in our city population with their death and recovery rates. Even then we cannot eliminate such cases as are thrown upon us from New York to Florida, and from the west. Chicago being a great railroad center, whose lines traverse vast regions where there are no asylums, from whence many of our cases are sent to us.

Dr. Bluthardt strove for four years to better the detention jail for insane in the city, and, while he has succeeded in improving matters, there remains a great deal undone.

TABLE XIV.

SHOWING THE PSYCHOSES OF PATIENTS ADMITTED DURING FISCAL YEAR ENDING AUGUST 31, 1883, RE-ADMISSIONS INCLUDED.

| | Males | Females | Total |
|---|---|---|---|
| Melancholia | 22 | 146 | 168 |
| Mania | 122 | 36 | 158 |
| Katatonia | 2 | ........ | 2 |
| Stuporous Insanity | 1 | ........ | 1 |
| Primary Mental Deterioration | ........ | 1 | 1 |
| Hysterical Insanity | ........ | 5 | 5 |
| Alcholic " | 7 | 2 | 9 |
| Epile;tic " | 14 | 5 | 19 |
| Recurrent " | 10 | 1 | 11 |
| Circular " | 1 | ........ | 1 |
| Imbecility | 6 | 1 | 7 |

TABLE XIV.—Continued.

| | Males | Females | Total |
|---|---|---|---|
| Idiocy | 1 | ......... | 1 |
| Monomania | 7 | 2 | 9 |
| Senile Dementia | 5 | 7 | 12 |
| Hebephrenia | 3 | ......... | 3 |
| Paretic Dementia | 6 | 1 | 7 |
| Delirium Grave | ......... | 1 | 1 |
| Terminal Dementia | 18 | 1 | 19 |
| Undiagnosed | 7 | 6 | 13 |
| | 232 | 215 | 447 |

It is not at all to the credit of this commonwealth that the laws relating to the insane befit a stage of barbarism, nor can the people of Chicago be complimented for having allowed Dr. Spray to fight single-handed for the exaltation of this asylum to a respectable medical plane.

---

ARTICLE II.

